# Evaluation of the quality and accuracy of breast cancer knowledge among persian language websites

**DOI:** 10.1186/s12913-022-08966-4

**Published:** 2022-12-20

**Authors:** Sadaf Alipour, Shekoofeh Nikooei, Reza Hosseinpour, Mohammad Javad Yavari Barhaghtalab

**Affiliations:** 1 Breast Disease Research Center, Cancer Institute, Tehran University of Medical Sciences, Tehran, Iran; 2 Department of Surgery, Arash Women’s Hospital, Tehran University of Medical Sciences, Tehran, Iran; 3 Student Research Committee, Yasuj University of Medical Science, Yasuj, Iran; 4 Department of General Surgery, Shahid Jalail Hospital, Yasuj University of Medical Sciences, Yasuj, Iran

**Keywords:** Health knowledge, Breast cancer, Internet, DISCERN instrument

## Abstract

**Introduction:**

The internet has become a powerful worldwide information source that revolutionized access to knowledge, especially in the fields of health and medicine (health knowledge). Therefore, providing high-quality, accurate, reliable, and relevant information on dependable websites is a possible way of providing the patient with needed information and, thus, achieving the benefits of informed patients regarding outcomes. This study aimed to evaluate the quality and accuracy of breast cancer knowledge among Persian language websites.

**Methods:**

Two search engines were searched in the Persian language about breast cancer. The first 30 websites were selected for further evaluation based on the completeness, correctness, transparency, and accessibility of health knowledge. The DISCERN instrument was used to assess the quality of the Persian language websites on this issue.

**Results:**

Among the 30 websites, about 23% of websites provide completely correct information and about 30% provide mostly correct information. Sixty percent of the websites provided author information, and 46% of them had a healthcare professional or expert as the author. Sixty percent of the websites stated the creation date on the pages, while 40% of them did not provide any health knowledge. Scores on accessibility were always easy for most of the websites. Based on the quality rating system of DISCERN, about 60% of the websites were presented as very poor.

**Conclusion:**

Website rankings enable healthcare professionals to identify and signpost patients to reliable up-to-date websites to ensure that patients receive high-quality knowledge. This review has provided evidence of inadequate and inaccurate health knowledge about breast cancer on the Persian language websites. This issue requires further investigation to understand the barriers and solutions available to provide reliable information about breast cancer and how this information affects the patient's outcomes.

**Trial registration:**

The project was found to be in accordance with the ethical principles and the national norms and standards for conducting research in Iran with the approval ID and date of IR.TUMS.IKHC.REC.1399.379 and 2021–01-01 respectively, and is registered with research project number 49890 in the Vice Chancellor for Research and Technology Development of Tehran University of Medical Sciences, Tehran, Iran.

URL: https://ethics.research.ac.ir/EthicsProposalViewEn.php?id=170978.

**Supplementary Information:**

The online version contains supplementary material available at 10.1186/s12913-022-08966-4.

## Introduction

In recent years, the internet has become a powerful worldwide information source that revolutionized access to knowledge, especially in the fields of health and medicine [1]. Many patients and their relatives are turning to the internet for health knowledge. About 49.2% of the world's population uses the internet with more than 57.4% in the Middle East and 72% in the United States [2, 3]. Estimation of a meta-analysis study’s results showed a mean health literacy of 59.96, with a confidence interval (CI) of 57.01 to 62.90 in the Iranian population [4]. Among internet users, 59% have searched for health-related and medical information that 66% of them search for a specific disorder or disease, and 56% suggested searching for treatments [5]. However, this information could be inaccurate or incomplete. The accuracy and quality of medical information on the websites influence patient decisions concerning their treatment. On one hand, some websites’ health knowledge could be confusing to the audience or may exploit vulnerable patients by selling useless products or harmful devices [6, 7]. On the other hand, misinformation can delay timely action to control and treat the disease, or it may lead to the use of unverified therapies instead of standard ones [8]. This is especially true for information about cancer in general and breast cancer in particular. Among different types of cancer, breast cancer is the most common one with the deadliest malignancy among females comprising 18% of all women's cancer, both in developed and developing countries and Iran is not an exception [9]. According to the world health organization (WHO), breast cancer is classified as one of the most worrying factors in women's health [10] with significant mortality, accounting for approximately 15% of all female cancer deaths [11].

In order to diagnose and prevent breast cancer before the presentation of symptoms, mammography and ultrasonography should be done as the screening method in asymptomatic and high-risk patients [12, 13]. Treatment of breast cancer usually includes mastectomy and axillary lymph node dissection depending on the stage of the breast cancer. Radiation therapy, chemotherapy, hormone therapy, and targeted biological therapy can also be used depending on the stage of cancer and its biological characteristics [14–16].

Various studies have shown that health knowledge about breast cancer can inform women about their disease and help them to make better decisions about treatment and care [17, 18]. In addition, according to the stage of breast cancer, a person needs different information [14]. Although patients can access physicians, pharmacists, and other healthcare providers, they may prefer to use the internet in some conditions including 1) lack of enough time, 2) the importance of the issue, and 3) distrust healthcare providers or the healthcare system. As a result, medical information needs to be properly processed and organized while searching so that the users can meet their specific needs [19].

The aims of this study, therefore, were to evaluate the completeness, correctness, accessibility, and transparency of breast cancer information provided to patients on the internet. Besides, the DISCERN instrument was used to assess the quality of the Persian language websites on this issue.

## Methodology

### Study aims

In this article, a cross-sectional study was designed to investigate the accessibility, transparency, completeness, depth, and accuracy of Persian language breast cancer websites. These criteria are based on the criteria described by Ream et al. [20], Nilsson-Ihrfelt et al. [21], and Silberg et al. [22] as well as the DISCERN instrument developed by the Department of Public Health and the Department of Primary Health Care at the University of Oxford [21–24]. These assessments were performed under the supervision of an oncology surgeon.

### Search strategy

**Fig. 1:**
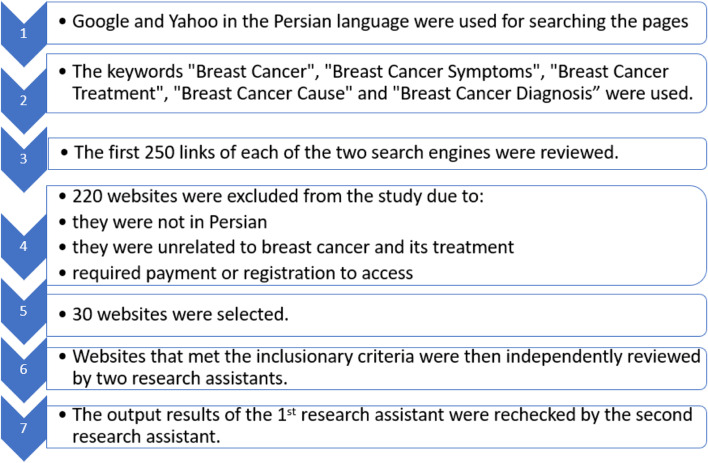
Search strategy: a PRISMA style flow chart

The search for websites was conducted in Iran in June 2021 using Google Chrome version 90.0.4430.212, on the two following engines: Google (http://www.google.com), and Yahoo (http://www.yahoo.com). Google and Yahoo are the topmost search engines in Iran according to Alexa ranking [25]. To add, as the query was performed in Iran and therefore the Google installation primarily searches for pages in the Persian language. The keywords used were: “Breast Cancer”, “Breast Cancer Symptoms”, “Breast Cancer Treatment”, “Breast Cancer Cause” and “Breast Cancer Diagnosis”. The first 250 links of each of the two search engines were reviewed. All websites included health knowledge on breast cancer, such as risk factors, symptoms, diagnosis, treatment, and psychological support in the Persian language. On the other hand, the websites were excluded from the study if they were not in Persian and if they were unrelated to breast cancer and its treatment. Also, the websites that required payment or registration to access were excluded. Websites that met the inclusionary criteria were then independently reviewed by two research assistants. The output results of the 1^st^ research assistant were rechecked by the second research assistant. Both of them were experts in this field. If there were disagreements between the two research assistants who ranked the websites, the oncology surgeon would resolve them. After applying restrictions based on inclusion and exclusion criteria, thirty websites remained. A PRISMA-style flow chart is shown in Fig. 1 for the search strategy. Selected websites with their website addresses are presented in Table 1.

**Table 1:** Selected websites with their website addresses

	**Websites**	**Website address**
1	darmankade	https://www.darmankade.com/
2	vclinic	https://vclinic.io/blog/
3	namnak	https://namnak.com/
4	ibcrc	http://ibcrc.ir/
5	ncii	http://ncii.ir/
6	bcpi	http://bcpi.ir/
7	vitrinmed	https://www.vitrinmed.com/
8	beytoote	https://www.beytoote.com/
9	dr-kaviani	http://www.dr-kaviani.com/
10	pezeshkat	https://pezeshket.com/
11	zoomlife	https://zoomlife.ir/health/diseases/
12	fa.parsiteb	https://fa.parsiteb.com/
13	drdr	https://drdr.ir/
14	madarsho	https://madarsho.com/
15	tebyan	https://article.tebyan.net/
16	iranpath	http://iranpath.org/
17	rooziato	https://rooziato.com/
18	pardiscancer	https://pardiscancer.com/
19	sadrasono	http://sadrasono.com/
20	ircancercenter	http://www.ircancercenter.com/
21	chetor	https://www.chetor.com/
22	drzakerin	https://www.drzakerin.com/Breast-Cancer-Symptoms
23	dr-hashem	https://dr-hashem.com/
24	drziaei	http://www.drziaei.com/
25	arioclinic	https://arioclinic.com/bcc/
26	lafarrerr	https://lafarrerr.com/blog/cancer-symptoms/
27	salamatbank	http://salamatbank.com/disease/description
28	jameesalamat	https://jameesalamat.com/
29	cnin	http://cnin.ir/Cancer-Diagnosis-and-Prognosis.as
30	iranbmemag	https://iranbmemag.com/

### Completeness of breast cancer knowledge

Nilsson-Ihrfelt et al. defined completeness based on the evaluation of the quality dimension and level of detail in 2004. As many as ten questions were asked in the quality measurement section as risk factors, screening and mammography, surgical treatment, chemotherapy, radiotherapy, hormonal treatment, other types of pharmaceutical treatments, breast reconstruction, complementary medicine, and emotional and/or psychological support. knowledge was scored based on one mark for each criterion (Total = 10). A score was then determined for the depth of knowledge with 0 attributed to ‘none’, 1 ‘minimal’, and 2 ‘more than minimal’. A score ranging from 0 to 30 was generated after summing the scores. The subject is to be examined not mentioned at all in the category of ‘none’. A brief discussion of the subject is needed in the categorization of ‘minimal’ and a detailed discussion of the subject is needed in the categorization of ‘more than minimal’ [3, 26].

### Correctness of knowledge

Online sources were assessed by comparison with recognized peer-reviewed sources of information named National Institute for Health and Clinical Excellence (NICE) guidelines on breast cancer. The sources used were the Royal College of Surgeons in Ireland (RCSI) breast cancer management guidelines, Surveillance, Epidemiology, and End Results (SEER) program for breast cancer survival, and the American Joint Committee on Cancer (AJCC) staging guidelines for breast cancer. The correctness of knowledge was characterized as ‘mostly incorrect’, ‘mostly correct’ and ‘completely correct’ [2, 3, 26].

### Accessibility

This criterion examined how easy it was to navigate around each website and find the information required on breast cancer while maintaining the simplicity of technology, operation, or format. Accessibility is rated with three items "Yes Always", "Yes Sometimes" and "No" [3, 26].

### Transparency of knowledge

Silberg et al. formulated the transparency of knowledge based on authorship, attribution, up-to-datedness, and disclosure in 1997, and this formula evaluates the transparency, honesty, authority, privacy, protection, and updating of information per website. The score for proof of authorship identity ranges from 0 to 8, which allows the user to easily check the reliability of the authors’ credentials. For attribution, scores for proof of the source of information range from 0 to 7. For references, we checked the credibility of the source. We determined the up-to-datedness of the site and scored each site from 0 to 4. For disclosure, honesty about funding and the use of personal information scored 0 to 5 [2, 3, 26].

### DISCERN instrument

We chose the DISCERN instrument because it is the first standardized quality index of consumer health knowledge that can be used as a critical appraisal tool to evaluate health knowledge by health professionals, patients, and the general population. The DISCERN questionnaire process is comprised of 15 questions plus an overall quality rating in three sections. The first Section contains 1–8 questions addressing reliability; questions 9–15 focus on the treatment information on the website, and question 16 addresses the overall quality score of the websites.

In Introduction section, the questions are about the reliability of the publication, the clarity of the aims, relevancy, clarity of the sources of information that were used to compile the publication (other than the author or producer), clarity of the information used or reported in the publication, balanced and unbiased information, providing details of additional sources of support and information, and referring to the areas of uncertainty. In Methodology section, the questions are about the quality of information on treatment choices, describing how each treatment works, describing the benefits of each treatment, describing the risks of each treatment, describing what would happen if no treatment is used, describing how the treatment choices affect the overall quality of life, clarity that there may be more than one possible treatment choice, and providing support for shared decision-making. In Results section, the overall rating of the quality of the publication as a source of information about treatment choices was done [27–29]. In each question, a score of 5 is assigned to the highest option and a score of 1 is related to the lowest with a maximum score of 80 (Table 2). After scoring, each website is ranked according to Table 3.

**Table 2:** DISCERN rating system for each question

**No**		**Partially**		**Yes**
1	2	3	4	5

**Table 3:** DISCERN benchmark ranking system

Category	Score
Excellent	68–80
Good	55–67
Fair	42–54
Poor	29–41
Very poor	16–28

### Data analysis

Scores were summed up in Excel. Scores for each quality criterion (completeness, transparency, and usability) were attained. In addition, the scores for each transparency attribute (authorship, attribution, up-to-datedness, and disclosure) were calculated then. Scores related to DISCERN criteria were also analyzed and presented according to the available rankings.

## Results

The outcomes of the website evaluation are presented by quality criteria. Performance is reported for all 30 websites, as well as by DISCERN instrument. As depicted in Table 1, among 250 searched links (50 links for each word), 30 websites were selected for evaluation. Websites of breast cancer knowledge were next presented based on criteria such as completeness (Table 4), transparency (Table 5), and quality (Table 6).

**Table 4:** Completeness, depth, and correctness of breast cancer websites

	**Completeness**^**a**^		**Depth**^**a**^			**Correctness**^**a**^		
Topics (%)	**Yes**	**NO**	**No info**	**Min info**	**Max info**	**Mostly not**	**Mostly**	**Completely**
Risk factors	17 (56%)	13 (44%)	1(3%)	9 (30%)	7 (23%)	1 (3%)	9 (30%)	7 (23%)
Screening and mammography	14 (46%)	16 (54%)	0	10 (33%)	4 (13%)	1 (3%)	7 (23%)	6 (20%)
Surgical treatment	17 (56%)	13 (44%)	1 (3%)	13 (44%)	3 (10%)	2 (6%)	10 (33%)	5 (16%)
Chemotherapy	15 (50%)	15 (50%)	1 (3%)	13	1 (3%)	3 (10%)	7 (23%)	5 (16%)
Radiotherapy	11 (36%)	19 (64%)	1 (3%)	9 (30%)	1 (3%)	4 (13%)	4 (13%)	3 (10%)
Hormonal treatment	10 (33%)	20 (67%)	0	9 (30%)	1 (3%)	3 (10%)	4 (13%)	3 (10%)
Other pharmaceutical treatments	2 (6%)	28 (94%)	0	2 (6%)	0	0	2 (6%)	0
Breast reconstruction	0 (0%)	30 (100%)	0	0	0	0	0	0
Complementary medicine	1 (3%)	29 (97%)	0	1 (3%)	0	0	0	1 (3%)
Emotional/psychological support	3 (10%)	27 (90%)	0	0	3	0	0	3 (10%)

^a^ Data present as the sum of the individual score

**Table 5:** Transparency of breast cancer websites

Transparency	Yes for all	Yes for some	No
*Authorship*			
Is there a disclosure of authorship?	18(60%)	0	12 (40%)
Is there a disclosure of the authors’ credentials?	15(50%)	0	15 (50%)
Is the author a healthcare professional/expert?	14(47%)	3(10%)	13 (43%)
Are the credentials verifiable?	23(76%)	2(7%)	5 (17%)
Are the author’s contact details provided?	15(50%)	0	15 (50%)
*Attribution*			
Is the source of information clear?	8 (27%)	0	22 (73%)
Are references given?	4 (13%)	0	26 (87%)
Is opinion stated as such?	5 (17%)	0	25 (83%)
Are any working external links provided to scientific reference material/studies?	3 (10%)	2 (7%)	25 (83%)
*Currency*			
Is the creation date of each page given?	18 (80%)	0	12 (40%)
Is the date of the last update clearly stated?	5 (17%)	1 (3%)	24 (80%)
Has the site been updated within the last 4 months?	3 (10%)	1 (3%)	26 (87%)
*Disclosure:commercial interests*			
If the site is commercial, is the source of funding clearly stated?	7(23%)	2(7%)	21(70%)
Is the site selling a product?	3(10%)	0	27(90%)
Does the site carry adverts?	10(33%)	0	20(67%)
Does the site allow pop-ups?	10(33%)	0	20(67%)
Is the privacy policy easy to find and clear?	10(33%)	9	11(37%)
Is personal information disclosed to the site sold to other organizations?	3(10%)	0	27(90%)

**Table 6:** Quality of breast cancer websites using DISCERN

**Websites**	DISCERN **score**	DISCERN **quality rating**
darmankade	30	Poor
vclinic	22	Very poor
namnak	26	Very poor
ibcrc	30	Poor
ncii	68	Excellent
bcpi	28	Very poor
vitrinmed	34	Poor
beytoote	32	Poor
dr-kaviani	54	Fair
pezeshkat	46	Fair
zoomlife	32	Poor
fa.parsiteb	22	Very poor
drdr	24	Very poor
madarsho	26	Very poor
tebyan	26	Very poor
iranpath	44	Fair
rooziato	44	Fair
pardiscancer	20	Very poor
sadrasono	24	Very poor
ircancercenter	28	Very poor
chetor	26	Very poor
drzakerin	22	Very poor
dr-hashem	22	Very poor
drziaei	26	Very poor
arioclinic	24	Very poor
lafarrerr	18	Very poor
salamatbank	28	Very poor
jameesalamat	34	Poor
cnin	32	Poor
iranbmemag	28	Very poor

### Completeness and depth of knowledge

The results of breast cancer information on selected websites in terms of completeness, depth, and correctness, are presented in Table 3, showed that a large number of websites have information about risk factors (56%), surgical treatment (56%), chemotherapy (50%), screening and mammography (46%), radiotherapy (36%), hormone therapy (33%). However, some websites provided information on other types of pharmaceutical treatments (6%), complementary medicine (3%), and emotional and psychological support (10%). On the other hand, none of the websites have any information about breast reconstruction (0%).

Regarding the depth of breast cancer knowledge on websites, the websites were categorized into three levels: 1. No information, 2. Minimum information, 3. Maximum information. The maximum information is related to breast cancer risk factors (23%), although most of the websites displayed minimal information about mammography and screening (33%) and surgical treatment (44%). Other websites were not at a good level with the depth of information. (Table 3).

### Correctness of knowledge

The correctness of knowledge was categorized into three levels: mostly not correct, mostly correct, and completely correct. Evaluation of the correctness depicted that about 23% of websites were estimated to give completely correct knowledge and about 30% provide mostly correct information concerning the risk factors of breast cancer. Regarding screening and mammography, 20% of the websites were completely correct and the remaining 23% were mostly correct. The least amount of correct knowledge about radiotherapy followed by hormonal treatment for breast cancer was given (Table 3).

### Accessibility

In this context, the websites had good accessibility in which access to 86% of the websites was easy, while only 13.3% of the other websites were sometimes unavailable and 0% of the websites were out of reach.

### Transparency

Transparency of knowledge on breast cancer-related websites, including authorship, attribution, up-to-datedness, and disclosure, is shown in Table 5.

#### Authorship

Sixty percent of the websites provided author information while 40% of them did not provide any author information. And 46% of them had specialist or professional healthcare, while 43% of the websites had no professional or specialist author. One percent of the websites were provided by a specialist. Interestingly, 50% of the websites provide the author’s contact information while 50% of them did not provide such details (Table 4).

#### Attribution

The main point in this part is whether the sources of information provided are real, clear, and identifiable. 26.6% of the websites cited valid sources, while 73.3% of them did not provide any accurate sources of information. On the other hand, just 13.3% of the websites provided references (Table 5).

#### Up-to-datedness

Assessment of the relevance and up-to-datedness of each website showed that 60% of the websites stated a specific date of page creation, while the rest, 40% of the websites did not provide any information about the production date of the content (Table 4). 16.6% of the websites clearly stated the date of the last update of the content, and 80% of the websites did not mention any information. The overall scoring of this feature showed that almost half of the websites do not accomplish well to provide up-to-date content.

#### Disclosure

The disclosure measured each website’s openness about funding and the use of personal information. Approximately 23% of websites provided clear information about their source of funding, 6% of the websites provide minimal information and 70% of them provide specific information from their source. Importantly, financial sources or conflicts have not been disclosed. 1% of the websites sold products while most sites (90%) did not sell any products on their website. About 33% of the websites carried advertisements. In 33% of the websites, it was possible to pop up (open a window on the page). The privacy policy was clear for about 33% of the websites. However, only 1% of the websites allow the sale of website information to other organizations.

### Websites assessment based on DISCERN questionnaire

The results of website quality assessments evaluated based on DISCERN questionnaire are presented in Table 5. None of the surveyed websites reached a maximum score of 80. The only website that was ranked as excellent in the range (68-80) was the National Center on Intensive Intervention's (NCII) mission with a score of 68. Similarly, none of the websites were in the good range (6756). Approximately 13% of the websites scored with an average score (54-54) followed by 23.3 % of the websites with poor criteria (41-29). According to the DISCERN quality rating system, 60% of the websites were rated as very poor, which indicates that according to the DISCERN benchmark, most of the websites reviewed have poor performance and quality.

## Discussion

The main goal of this study was to evaluate the accuracy and quality of the Persian language breast cancer websites available on the Internet. These websites were evaluated for completeness, correctness, transparency, and accessibility, and the websites were also scored using the DISCERN criteria. The Google and Yahoo search engines were used to search the keywords “breast cancer”, “breast cancer symptoms”, “breast cancer treatment”, “breast cancer cause” and “breast cancer diagnosis” and 30 websites containing information related to breast cancer were selected for further evaluations.

We checked all 30 websites for their completeness and correctness, which showed different results for every item which includes; risk factors, screening and mammography, surgical treatment, chemotherapy, radiotherapy, hormone therapy, other medications, reconstruction methods, complementary medicine, and emotional or psychological support. Risk factors, surgical treatment, and chemotherapy were the three main topics that were covered in the reviewed websites (about 54% of all websites). Additionally, no information was found about breast reconstruction on any of the websites, and this might be due to cultural reasons. Topics that were minimally addressed were complementary medicine (3%), and other medications (2%), followed by emotional and psychological support (10%).

Risk factors and mammography screening were the most correct subjects (23%), while the maximum score for depth of information was related to breast cancer risk factors (23%), and then to a lesser extent, items such as mammography and screening and surgical treatment could be seen. A study conducted by Alnaim in 2019 declared that the information about breast cancer published in Arabic on these websites included risk factors (93%), screening and mammography (93%), surgical treatment (93%), chemotherapy (89%), radiotherapy (93%) and complementary drugs (0%). In addition, about 67% of the evaluated websites provided completely correct information [26]. However, in comparison with this study, the Persian language websites had lower completeness and depth.

In the current study transparency of Persian language websites about breast cancer was calculated using various criteria such as authorship, attribution, up-to-datedness, and disclosure of information. The summary of the scores showed that most of the websites contain relatively poor information for attribution, as the World Health Organization (WHO) revealed that websites that provide information on breast cancer had a relatively lower score compared to websites with other types of information [20]. According to our research, 60% of the websites provided author information, and 46% of them had a healthcare professional or expert as the author. While other studies declared that, only 35% of websites were authored by a healthcare professional [25]. Therefore, we are more likely to expect the websites to provide better and more accurate author information, as well as the same content provided by a healthcare professional.

According to the accessibility, in our study, 86% of the websites were easy with good accessibility. Akuoko et.al. studied the quality of breast cancer information on the internet in Africa and reported a moderate to high score for accessibility to these kinds of information [30].

Meric et.al has shown that the quality of websites by using the DISCERN instrument, demonstrated that most websites had poor and the rest had average quality [31, 32]. Similarly, in our study, most websites did not meet the expectations to provide quality information about breast cancer to an extent that 60% of websites were rated as very poor based on the DISCERN quality rating system.

The main advantage of the internet today is that, unlike traditional sources, it provides an easy and free way to update people’s knowledge. Many websites now incorporate Web 2.0 technologies to make more dynamic content. Also, it provides access to social networking sites, chats forums, live chats, and blogs that can promote and update one’s information in a short time. However, it is noteworthy that business websites and organizations need better governance in providing information [2, 20].

One of the most critical subjects which cannot be neglected is that on the internet space, everyone with every state of knowledge can share and present information about breast cancer. Hence, there should be supervision of the presented knowledge and the authors [32]. Therefore, the quality and accuracy of breast cancer information should be constantly improved and updated by professional healthcare or author, in order to be introduced to the patients as a reliable source to promote their information about breast cancer, by their physicians [5, 33]. We also suggest that physicians, webmasters, and authors should be more accurate in producing knowledge about breast cancer and all of this knowledge should be reviewed by a physician and oncologist before publication. Last but not least, high-quality, accurate, reliable, and relevant content on websites is an accessible and simple method for patients to provide the necessary information at the right time and relieve their anxiety so, it can help them to make their treatment decisions with more awareness.

HON (Health On the Net) Foundation issued a code of conduct (HON code) for medical and health websites to address the reliability and usefulness of medical information on the Internet. The principles of the HON code are authority, complementarity, confidentiality, attribution, justifiability, transparency of authorship, transparency of sponsorship, and honesty [34, 35]. In this paper, depth of knowledge, the correctness of the information, accessibility, and up-to-datedness were the terms that were more discussed compared to the HON.

## Conclusion

Websites providing breast cancer information in the Persian language should be improved in terms of completeness, correctness, transparency, and accessibility. There are a limited number of websites that have reliable information about breast cancer in Persian, of which the National Center on Intensive Intervention (NCII) website was the best. Current research and all previous research show that most websites do not provide users with complete and correct information about breast cancer. Although the number of patients has increased, in most cases websites haven’t updated their content. Also, access to accurate information on the internet will help patients decide on their treatment methods. Generally speaking, it is the right of every patient to have access to complete, accurate, and reliable health information in a native language. Many aspects of the topics covered by this article require further investigation to understand barriers and solutions available to provide reliable information about breast cancer and how this information can affect the patient's outcomes.

The key message to the international community of informaticists, web designers, and patient advocacy groups is to build up accurate information on the internet and improve patient educational materials by using healthcare professionals in breast surgery, breast oncology, breast nursery, and midwifery, and giving the National Center on Intensive Intervention (NCII) website as the best one of its kind in rural and urban health centers in family health programs.

## Supplementary Information

**Additional file 1.**

## Data Availability

The raw data is provided in a supplementary file and the datasets used and/or analyzed during the current study is available by request to the corresponding author.

## Abbreviations

AJCC: American Joint Committee on Cancer
HON: Health on the Net
NCII: National Center on Intensive Intervention
NICE: National Institute for Health and Clinical Excellence
SEER: Surveillance Epidemiology and End Results
WHO: World Health Organization
